# Participatory health research and promotion with migrant communities in Germany: a scoping review

**DOI:** 10.3389/fpubh.2025.1585178

**Published:** 2025-09-12

**Authors:** Hanna Luetke Lanfer, Janna Landwehr, Doreen Reifegerste

**Affiliations:** School of Public Health, Bielefeld University, Bielefeld, Germany

**Keywords:** participatory health research, migrant health, peer research, health equity, reflexivity, scoping review

## Abstract

**Introduction:**

Participatory health research and promotion aims to foster inclusive knowledge production and address health inequities. Migrant communities, given their diverse backgrounds and health needs, are regularly engaged in PHR/P. However, the extent and ways of their participation across research phases and the integration of critical reflections in published studies from Germany remain underexplored.

**Methods:**

This scoping review followed Arksey and O’Malley’s framework and adhered to PRISMA guidelines. Four databases were systematically searched for eligible studies. A total of 17 publications representing 13 projects were included and analysed using a structured codebook.

**Results:**

Migrants’ participation was described unevenly across different phases of the research process, with more frequent engagement in operational aspects such as data collection than in research design, analysis, or dissemination. Reflexivity was inconsistently reported. While some studies provided theoretical reflections on participation, explicit links to how these reflections shaped to research practices were often missing. Reflections also focused more on methodological and external challenges than on power dynamics, ethics, or researcher positionality.

**Discussion:**

Since our analysis is based on published accounts, the extent to which participation and reflexivity were practiced beyond what was documented remains unclear. More systematic documentation of participatory processes and reflexivity would enhance transparency, reflect the complexities of PHR/P with migrants more deeply, and inform future practice.

## Introduction

In Germany, nearly a quarter of the total population has a migration background Statistisches (1). This includes first- and second-generation migrants from different regions of the world, with varying legal statuses, socio-economic conditions, and linguistic diversity as well as diverse health perceptions, risk factors, and access to healthcare (2, 3); Statistisches (1, 4). Thus, Germany is characterized by a highly diverse and continuously growing migrant population and one of the main countries of immigration in Europe.

Because understandings of the relationship between migration experiences and health remain inadequate and fragmented, migrants have become a focal point for participatory health research globally, including Germany (5–8). Participatory approaches foster collaboration among academic researchers and affected communities and thereby aim for a deeper understanding of sociocultural contexts relevant for health, bridging the gap between academic knowledge and everyday realities and promoting health equity (9, 10). Although becoming increasingly common in health promotion and health research, the versatility of participatory approaches comes with their own challenges (11, 12). These include debates on classifications and rigor (13) as well as tensions between ideals of co-creation and realities shaped by representation, resources, group and power dynamics (6, 14). These challenging dynamics are particularly pronounced in research with migrant communities, where structural inequalities, socio-economic exclusion, legal uncertainties, and other barriers (such as language, cultural norms, and competing priorities) can intersect with participatory processes in ways that shape both the extent and nature of involvement (6, 7, 15, 16).

While Germany’s participatory health research landscape is expanding [see (17)], there is no comprehensive overview of how migrant communities are engaged in participatory health approaches – especially who participates, during which phases of the research process, and how reflections on participation are reported. To address this gap, our scoping review systematically identifies and synthesises published research on participatory approaches with migrant communities in Germany.

### Conceptual framework of participatory approaches in health

There is a wide spectrum of participatory approaches with diverse, sometimes overlapping terminology (6, 8, 18). In this article, we focus on two forms of participatory approaches related to health: participatory health research (PHR) and participatory health promotion (PHP) that can be distinguished by their core objectives. PHR is designed to generate knowledge through systematic inquiry in close collaboration with the communities involved with the research process itself, often serving as a means of empowerment and social change (18). By contrast, PHP aims to improve health outcomes by co-creating, implementing, and evaluating interventions with communities, with research often a secondary rather than primary aim (19, 20). Given the broad terminology in participatory health approaches, we use the term participatory health research/promotion (PHR/P) to encompass both forms (21), acknowledging their shared emphasis on collaboration while recognizing their distinct orientations toward knowledge production or practical intervention.

PHR/P is rooted in the principles of collaboration, shared ownership, and the redistribution of power in research processes (22, 23). It challenges conventional research paradigms by emphasizing equity between academic and community partners, valuing experiential knowledge alongside scientific expertise (18, 21). It bridges research and practice, aiming to ensure that interventions reflect lived realities, while also empowering participants and strengthening local capacities (6, 24)

Working within an open and holistic paradigm, PHR/P embraces methodological diversity, allowing research approaches to be tailored to the specific needs and contexts of participating communities (21). The inherent flexibility of PHR/P is its hallmark, enabling it to adapt to diverse settings and participants’ needs. However, this adaptability also introduces challenges in defining how participation is structured and whose voices are represented (12, 24, 25). Moreover, with the increasing adoption of PHR/P, concerns have emerged regarding tokenistic and ritualistic participation, where involvement is superficial rather than substantive (6). While there is a range of existing frameworks to guide PHR/P, there is limited systematic analysis of how the principles of PHR/P translate into practice, particularly in Germany, where a large and diverse migrant population intersects with a federal health system, complex access pathways, and policy frameworks that differ from those of other countries (26).

Given Germany’s particular context as a major European country of immigration, this review examines how participation in PHR/P with migrant communities in Germany is described in publications. It addresses the overarching research question:

*How are participatory health research and promotion (PHR/P) with migrant communities in Germany described in the scientific literature?*

To address this question, we examine four sub-dimensions:

a. Stated health- and participatory-related objectives in PHR/P with migrant communities

PHR/P often combines health-related aims, such as improving health literacy, behavioral change, or access to services (25, 27), with participatory aims, including capacity building, empowerment, and challenging hierarchies in knowledge production (6, 21). While these objectives are interrelated, we explore how they are framed in the literature, and to what extent health impact or participatory processes are emphasized.

b. Described participants in PHR/P with migrant communities

PHR/P emphasizes collaborations and partnerships, with participant composition varying widely depending on the context and project (9, 28). Collaborations may involve academic researchers, non-academically trained co-researchers (hereafter referred to as “co-researchers”), such as migrants or representatives from participating communities, and other stakeholders like NGOs, healthcare providers, or policymakers (9, 18, 29). We examine how participant groups are described, whose voices are represented and whose are not, and how inclusion or exclusion is addressed in German studies.

c. Participation in research phases in PHR/P with migrant communities

PHR/P aspires to enable the most comprehensive participation from those individuals directly involved or impacted by the study (18). Ideally, co-researchers engage across all phases, from defining questions to sharing results. A range of scales or typologies [for an overview see (30)] have been developed to guide those conducting PHR/P, yet authors acknowledge that this level of participation is not always possible, taking into account factors such as distinct resource availability (e.g., time, financial means, research experience, legal uncertainties), administrative frameworks and what can and cannot be imposed on co-researchers such as migrants. The International Collaboration for Participatory Health Research (31) hence calls for optimum participation and using the potential of participation where and whenever possible. We examine how German studies describe participation in different research phases, and how participation is adapted to resources, contexts, and constraints.

d. Project-related reflections in PHR/P with migrant communities

Reflexivity is central to PHR/P, encouraging researchers to critically examine power dynamics, roles, and relationships within the research process (6, 18, 32, 33). Reflexivity fosters awareness of social positions, resource allocation, and methodological choices, which often become spaces where power is negotiated and redefined (32, 34). In the context of PHR/P with migrant communities, reflexivity takes on additional significance due to structural inequalities, legal uncertainties, and the precarious access to healthcare and research participation that many migrants face. We explore how studies reflect on these dynamics when working with migrant communities.

## Methodology

This scoping review was conducted following the methodological framework by Arksey and O’Malley (35). The reporting adheres to the PRISMA guidelines (36).

### Databases searches and search strategy

We searched for peer-reviewed and non-peer-reviewed (e.g., book chapters) literature with a Digital Object Identifier (DOI) published in English or German in four databases (Web of Science, PubMed, Psyndex, LIVIVO). We limited our search to literature published since 2000, as this period marks the increasing formalization of quality criteria in health promotion and the growing institutional recognition of participation in research and policy (37). The literature search was conducted on 12 of September 2023. Four search categories guided our search string: participatory research; migrant; Germany; and health. The categories and their synonyms were linked between each by the Boolean operators and/or as shown in Table 1.

**Table 1:** Overview of search categories and terms.

Category	Search terms
Participation	Participatory[Title/Abstract] OR “participatory health research” OR “participatory research” OR CBPR OR participation[Title/Abstract] OR “community based participatory research”[MeSH Terms] OR “Teilhabe” OR collaborat*[MeSH Terms]
Migration	Migran*[Title/Abstract] OR migration[Title/Abstract] OR refugee*[Title/Abstract] OR undocumented[Title/Abstract]
Country	“Germany”[MeSH] OR german*[Title/Abstract]
Health	“Health promotion”[MeSH Terms] OR “public health”[MeSH Terms] OR prevention[Title/Abstract] OR promotion[Title/Abstract] OR health[Title/Abstract]

The here displayed search string used for PubMed was adapted accordingly to match the indexing and search functionalities of the other databases. Moreover, we sent an email to the mailing list of PartNet, asking the researching community for further literature we might have missed during the database search.

### Selection of studies

Titles, abstracts and citations from all the searches were downloaded and duplicates removed manually by two reviewers. The records were screened by three reviewers (first author and two student assistants) against the inclusion and exclusion criteria (Table 2). To ensure accuracy and consistency in the coding process, records retrieved from one database (PSYNDEX, *n* = 68) were screened by all three reviewers; no disagreements were found. Following this, the abstracts from the remaining databases and responses from the mailing list of PartNet were screened by two reviewers each, i.e., one student assistant and the first author. Each record was marked as either “exclude,” “include” or “unsure” and disagreements were discussed between the reviewers. Similarly, publications that were considered eligible for the review were retained for a full-text review, each independently reviewed by a student assistant and the first or second author.

**Table 2:** Inclusion and exclusion criteria for PHR/P with migrants in Germany.

Criteria	Inclusion	Exclusion
Language	English or German	Publications in other languages
Publication date	From 2000 onwards	Published before 2000
Geographical focus	Studies conducted in Germany or focused on migrants in Germany	Studies outside Germany or lacking explicit focus on Germany
Focus population	Migrants, refugees, or undocumented populations in Germany	General populations without migrant-specific focus
Research approach	Author-reported participatory research (e.g., PHR, CBPR, community-based) or participatory health promotion	Non-participatory studies
Health focus	Health-related study	Non-health-related studies
Document availability	Full texts accessible via databases, institutional access or mailing list (PartNet)	Abstracts only or inaccessible texts

During the study selection process, we identified multiple publications related to the same projects. If these publications met the inclusion criteria and were deemed non-redundant—such as reporting on different aspects, methods, or outcomes of the project—they were included in the review. As a result, our final analysis included 13 distinct projects represented by 17 publications, with four projects contributing two publications each.

### Data extraction and analysis

The code book for our review was operationalized using the International Collaboration for Participatory Health Research “Characteristics of Participatory Health Research” (31) (see Supplementary file for the full code book). Code categories evolved around general publication descriptions (e.g., type of article, health topic, authorship, backgrounds of authors); theoretical conceptualization and terminology of the participatory study (e.g., use of theoretical models, participation framework, terminology for non-academic researchers); methodological approach (e.g., study design; recruitment, inclusion and exclusion criteria); participation in the research process (described phases of the research process with participation described); and reflections (e.g., effects of the participation design, ethical concerns, resources and context factors, positionality of the research team). The code book included closed and open categories for quantitative and qualitative data extraction. Due to our focus on how PHR/P is conducted with migrants in Germany, we neither focused on the results relating to the health topic of the publication, nor did we do a quality assessment of the overall study and rigor of the methods. This was an intentional decision, as the included studies were highly heterogeneous and our primary aim was to analyse participatory approaches rather than study quality or intervention effects.

Based on the codebook, an Excel sheet was created to tabulate the extracted data. The coding process involved both quantitative and qualitative approaches. While our coding framework was structured around the research dimensions (deductive), for open categories and in the dimension of reflections, we explicitly employed inductive coding to allow for the emergence of new themes and insights from the material. Three student assistants and two mid-career researchers (first and second author) participated in coding. To ensure consistency, a test coding phase was conducted at the start, during which all reviewers independently coded the same publication. Discrepancies were discussed, and the codebook was refined to ensure a shared understanding by clarifying the meaning of key terms, distinguishing between categories, and adding relevant examples where needed. For the main analysis, each included full text was coded independently by two reviewers—a student assistant and either the first or second author. The coded results were compared, and discrepancies were resolved through discussion between the two reviewers. Disagreements were classified as either human errors (e.g., inattention) or more systematic issues, which are reflected upon in the discussion section.

#### Quantitative coding

Closed categories in the codebook were coded numerically (e.g., 1 = Yes, 2 = Unclear, 3 = Not named), allowing for systematic analysis and the calculation of inter-rater reliability. Inter-rater reliability was determined using the ReCal2 tool (38) to compute Cohen’s kappa for each coding team. Kappa values varied slightly across teams, with an overall kappa value of 0.75, indicating substantial agreement. The distinction between the coding categories was carefully defined to ensure clarity and consistency. For example, “Yes” indicated explicit mention and elaboration, “Unclear” referred to instances where it was not evident whether a particular reflection was related to the project or theoretical aspects, or when certain concepts were mentioned without further explanation or connection to the project, and “Not Named” denoted the complete absence of reference to the category.

#### Qualitative coding

For open categories (often following quantitative coding of “Yes” or “Other,” see codebook), text passages relevant to the codes were copied into Excel cells. In addition, all passages were highlighted and marked in the original PDF files, ensuring traceability between the extracted data and the corresponding texts. Using the extracted text data, the first author conducted a qualitative content analysis, as described by Mayring (39), employing an inductive approach to identify emerging themes. This process involved iterative coding and the creation of new categories based on the data. For RQ 4, which focused on reflections, we combined deductive and inductive coding. Predefined categories (e.g., ethical aspects) were used to structure the analysis, ensuring coverage of key reflection areas. Within these categories, inductive coding was applied to capture additional nuances and emergent themes in how reflections were framed. This approach allowed for both systematic classification and deeper qualitative interpretation of how participatory processes were documented across the reviewed publications.

For projects with two publications, the analysis was conducted at the project level to avoid duplication. If a category was coded as “Yes” across all publications, it was recorded as a single “Yes.” If the category appeared in only one publication, this was still considered sufficient for a “Yes” designation. Conversely, if the category was absent across all publications, it was coded as “No.” This approach ensured that each project contributed a single, non-redundant entry to the overall analysis. Generative AI (ChatGPT from Open AI) was used for language refinement during manuscript preparation.

## Results

### Identification and selection of studies

**Figure 1:**
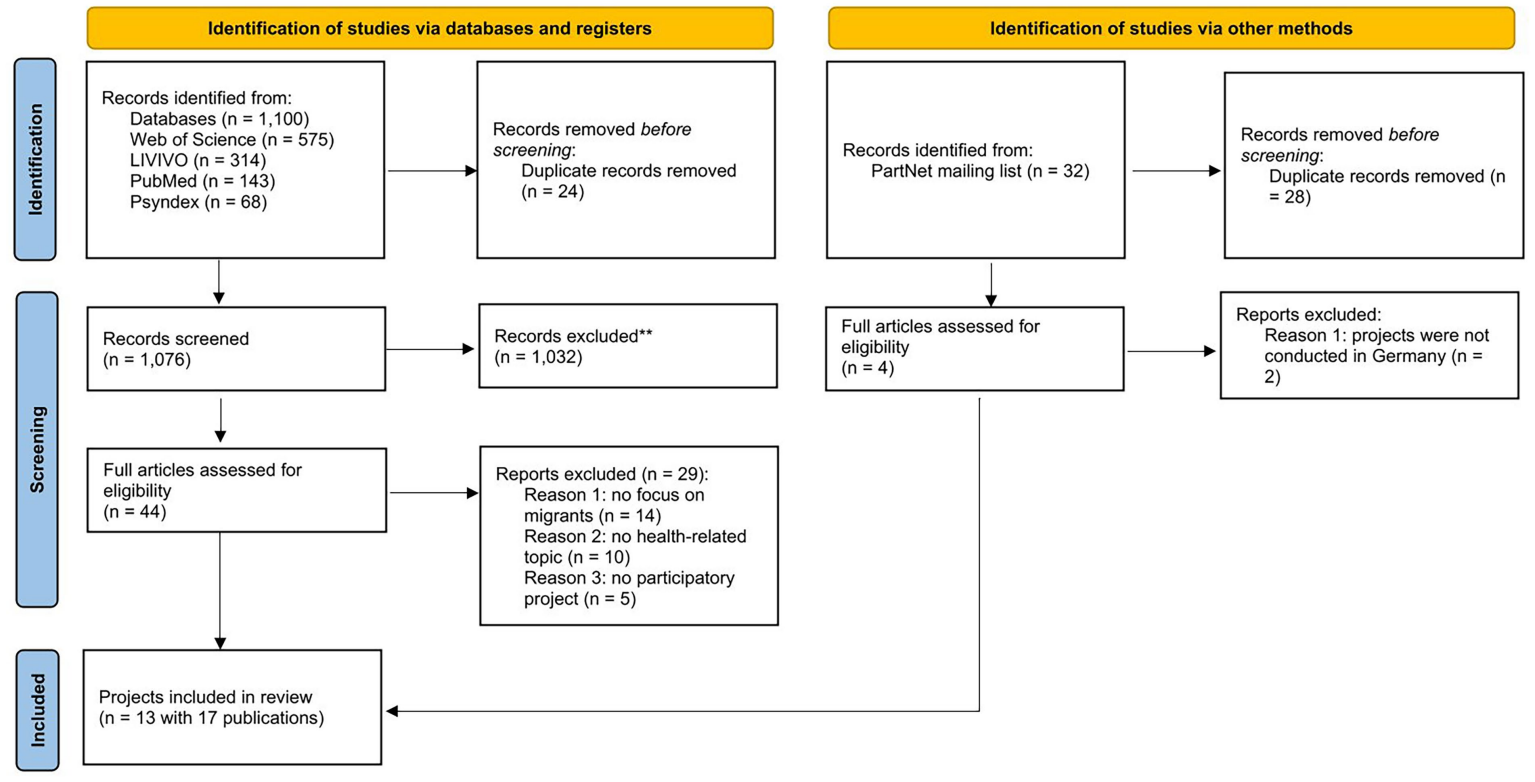
PRISMA-based flow diagram (65) depicting literature search, screening and selection processes.

The PRISMA flow diagram (see Figure 1) summarizes the processes used to identify and select studies for inclusion in this review. The search strategy identified 1,100 records from four databases (Web of Science, LIVIVO, PubMed, and Psyndex), along with 32 additional records received after our email to the PartNet mailing list. After the removal of 52 duplicates, 1,076 records remained for screening. Title and abstract screening led to the exclusion of 1,032 records, and 44 full-text studies were obtained for further review.

During the full-text review, 29 studies were excluded for the following reasons: no focus on migrants (*n =* 14), no health-related topic (*n =* 10), or no participatory project (*n =* 5). Ultimately, 13 distinct projects, represented across 17 publications, were included in the final analysis, with four projects contributing two publications each.

### Characteristics of included projects

The characteristics of the 13 included projects are presented in Table 3, with each project numbered for reference. Around one third of the projects (*n =* 5) were published between 2005 and 2014, while the majority of the included projects (*n =* 9) were published in the last 10 years. The majority of publications (counting *N* = 17) published their findings in peer-reviewed journals (*n* = 10), three in non-peer-reviewed journals, two as anthology contributions and one as a book chapter. The methodological landscape shows a predominant preference for qualitative (*n* = 7) and mixed-method designs (*n* = 5), with only one quantitative study (No. 7) (Table 3).

a. Stated health- and participatory-related objectives in PHR/P with migrant communities

**Table 3:** Characteristics of iNcluded Projects.

No	Author, year	Study design	Author identified type of research	Health-related objectives	Participatory-related objectives	Study setting	Main / Focus study population	Other stakeholder recruited	Authorship	Type of publication
1	Afeworki et al. (46)	Qualitative	Participatory health research	Identifying living circumstances / participation of persons with disabilities	Strengthening empowerment and social participation	Not named, interview participants chose comfortable and undisturbed places	Migrants with a disability	Professionals from social work and self-help / aid organizations for migration/ refugee or disability assistance; municipal institutions / boards	Academic researchers	Contribution to anthology
2	Evangelidou et al. (52)	Qualitative	Participatory health research	Improving healthcare access and quality of healthcare services for VMR (Vulnerable migrants and refugees) in Europe	Community empowerment and learning alliance	Not named	Vulnerable Migrants and refugees recently arrived (<5 Years in Europe)	Participants included migrants and refugees from outside EU countries as well as professionals from health and social sectors that work with migrants at the study sites.	Academic researchers, Stakeholders from non-academic institutions	Peer-reviewed article
3	Geldermann (45)	Qualitative	Participatory health research	Increasing digital health literacy	Participating in knowledge production	Community Center	Migrants from a specific district	No	Academic researchers	Peer-reviewed article
4	a) Herbert-Maul et al. (53) b) Fleuren et al. (54)	Mixed methods	Participatory health promotion	Promoting physical activity among people in difficult situations and the special challenges	Not named	Municipality	Women with low SES, migration background	Stakeholders from politics, practice, science, and local cooperation partners	Academic researchers	a) Peer-reviewed article b) Non peer-reviewed article
5	a) Kieslinger et al.(55) b) Kordel et al.(56)	Qualitative	Participatory health research	Exploring experiences of inclusion in rural areas	Encouraging participants to reflect and analyse their mobility biography	Community center	Refugees in rural areas	Professionals from local organizations act as gate keepers / co-researchers	Academic researchers	a) Peer-reviewed article b) Non peer-reviewed article
6	Koschollek et al. (57)	Mixed-Methos	Participatory health promotioN	Identifying health literacy and behavior regarding HIV and sexual health	Not named	Community center	Members of local African communities	Partner organizations with contact to local African communities	Academic researchers, Co-/Peer researchers	Non peer-reviewed article
7	Perplies et al. (58)	Quantitative	Participatory health promotion	Increasing immunization against measles, mumps and rubella	Strengthening self-responsibility and structures for self-help	Community Center	Migrants from a specific district / with low SES	Representatives of civil society organizations; municipal institutions	Academic researchers	Peer-reviewed article
8	Pallasch et al. (59)	Qualitative	Participatory health promotion	Strengthening mental health from refugees, reduce health inequalities, psychoeducation	Strengthening empowerment	Municipality, community- center	Migrants from Syria, Afghanistan	No	Academic researchers; Stakeholders from non-academic institutions	Peer-reviewed article
9	a) Salman (60) b) Salman et al. (61)	Mixed methods	Participatory health promotion	Strengthening empowerment and health literacy	Training mediators and capacity building for migrant communities	Municipality, community center, religious, social, cultural and educational institutions	Migrants from different countries (not specified)	No	Stakeholders from non-academic institutions	a) Peer-reviewed article b) Contribution to anthology
10	Samkange- Zeeb et al. (62)	Qualitative	Participatory health promotion	Communicating evidence- based health information by use of an “interactive health assistant” technology	Not named	Homes of participants or not specified self-chosen settings	Turkish migrants(born in Turkey/Turkish nationality)	No	Academic researchers; Stakeholders from non-academic institutions	Peer-reviewed article
11	Sauer et al. (47)	Mixed methods	Participatory health research	Evaluation of the situation and accommodation of unaccompanied refugee minors	Strengthening participation of minors in research	Municipality, community center	Refugee minors living in long- term residence	No	Academic researchers	Book chapter
12	Schön (63)	Qualitative	Participatory health promotion	Developing and increasing support offers for persons with disability and their relatives / mothers	Strengthening empowerment, self-organization, knowledge processes and application	Not named	Migrants with a disability and their relatives / mostly mothers	Tandem partners	Academic researchers	Non peer-reviewed article
13	a) Von Unger (18) b) Von Unger et al. (64)	Mixed-Method	Participatory health promotion	Developing HIV prevention programme and increasing cooperation between stakeholders	Strengthening participation and cooperation between stakeholders for HIV prevention	Not named	Migrants from African and European countries	Professionals from social work and aid Organizations; Representatives of project board	Academic researchers, Stakeholders from non-academic institutions, Co−/ Peer-researchers	a) Peer-reviewed article b) Peer-reviewed article

Across the 13 projects, slightly more than half were coded as PHP (*n* = 8), with the remainder as PHR (*n* = 5). Across projects, objectives (health- and participatory-related) often ran in parallel with their coding as either PHP or PHR. PHP projects commonly targeted behavior change or service uptake (e.g., vaccination uptake, No. 7), while PHR projects more often emphasized knowledge generation, capacity building, or community empowerment (e.g., community and stakeholder strengthening, No. 13). Health literacy was reported as an objective across both types (No. 3, 9, 10). An observable finding is the variability in the explicitness of participatory objectives: in three PHP projects (No. 6, 9, 10), participatory-related aims were only stated implicitly by describing community-facing activities elsewhere; this points to inconsistencies in how explicitly participatory intent is articulated at the level of reported objectives. Where health- and participatory-related objectives were reported together (e.g., No. 1, 3, 11, 12), the publications framed health improvement and participatory strengthening as mutually reinforcing (Table 3).

b. Participants described in PHR/P with migrant communities

Across studies, project teams consistently combined academic researchers with migrant co-researchers, and, variably, additional stakeholders (*n* = 7). Recruitment frequently added further criteria beyond “migrant,” for instance, low socioeconomic status (SES) or vulnerability (No. 2–4, 7, 11), country-of-origin (No. 8–9), disability status (No. 1, 11), or rural residence (No. 5–6). Stakeholders often functioned as gatekeepers, facilitators, or co-investigators, hence, their participation appeared purpose-built for community entry or logistics (No. 5, 7, 13) and were often included in projects working with ‘hard-to-reach-groups’, such as rural communities, low-SES groups, or migrants living with disabilities (Table 3).

c. Participation in research phases in PHR/P with migrant communities

Reported participation varied markedly across the six phases as participation peaked during instrument development and data collection and dropped sharply for analysis and outputs. In operationalization, migrants frequently piloted or adapted tools (e.g., translations, feedback; No. 1, 3–5, 7, 8, 10–11, 13). Data collection was also commonly participatory (No. 2, 3, 5–7, 9, 11, 13). By contrast, explicit involvement in analysis (No. 2, 12) and outputs/dissemination was less frequent; where reported, outputs included trainings, frameworks, and health materials (No. 3, 4, 10, 13). A noticeable aspect is the extent of “unclear/not named” reporting in early (conceptualization: No. 1–3, 5) and late phases (outputs: most projects except No. 13). This pattern indicates that participation often peaks where tasks are operational and time-bound, while roles in interpretive and disseminative tasks were either rare or insufficiently reported. Only two projects shared authorship in the reviewed publications with co-researchers (No. 6, 13) and five projects included the authorship of stakeholders from non-academic institutions (No. 2, 8–10, 13) (Table 4).

**Table 4:** Coded participation of focus population in different research phases.

No.	Author, year	Main/focus study population	conceptualization of research project (including defining research question and objective)	Operationalization of research instruments (including feedback to surveys, pilot testing and translations)	Data collection	Data analysis	Publications, policy papers, other outputs	Workshops, conferences	Other
1	Afework et al. (46)	Migrants with a disability	Unclear	Yes	Unclear	Unclear	Unclear	Unclear	N/A
2	Evangelidou et al., (52)	Vulnerable Migrants and refugees recently arrived (<5 Years in Europe)	Unclear	Unclear	Yes	Yes	Unclear	Unclear	N/A
3	Geldermann et al. (45)	Migrants from a specific district	Unclear	Yes	Yes	Unclear	Unclear	Unclear	Participation in training peer researchers and reflections
4	a) Herbert-Maul et al. (53) b) Fleuren et al. (54)	Women with low SES, migration background	Yes	Yes	Unclear	Unclear	Unclear	Unclear	Project framework development
5	a) Kieslinger et al. (55) b) Kordel et al. (56)	Refugees in rural areas	Unclear	Yes	Yes	Unclear	Unclear	Unclear	N/A
6	Koschollek et al. (57)	Members of local African communities	No	Unclear	Yes	Unclear	Unclear	Unclear	N/A
7	Pallasch et al. (58)	Migrants from a specific district / with low SES	No	Yes	Yes	Unclear	Unclear	Unclear	N/A
8	Pallasch et al. (59)	Migrants from Syria, Afghanistan	No	Yes	Unclear	Unclear	Unclear	Unclear	N/A
9	a) Salman (60) b) Salman et al. (10)	Migrants from different countries (not specified)	No	Unclear	Yes	Unclear	Unclear	Unclear	N/A
10	Samkangge- Zeep et al. (62)	Turkish migrants (born in Turkey/Turkish nationality)	No	Yes	Unclear	Unclear	Unclear	Unclear	Digital bilingual health assistant
11	Sauer et al. (47)	Refugee minors living in long- term residence	Yes	Yes	Yes	Unclear	Unclear	Yes	N/A
12	Schön (63)	Migrants with a disability and their relatives / mostly mothers	Yes	Unclear	Unclear	Unclear	Unclear	Unclear	N/A
13	a) Von Unger (18); b) Von Unger et al. (64)	Migrants from African and European countries	Yes	Yes	Yes	Yes	Yes	Yes	Digital materials

d. Described project-related reflections in PHR/P with migrants

We recorded whether reflections were explicitly reported and then inductively grouped the reported content (see Tables 5, 6). An observable finding is the concentration of reported reflections on methods (10 projects) and influence of external events (8 projects), while ethics (4 projects) and positionality (4 projects: No. 1, 2, 11, 13) appeared less often. Notably, methodological challenges in relation to different research phases were recurring topics. For example, mistrust towards research or institutions during recruitment (No. 1, 5), managing group dynamics (No. 4, 5, 8, 11) and difficulties with interpreter-mediated interviews during data collection were highlighted (No. 1, 5). Reflections on collaboration-related challenges (7 projects) were also common.

**Table 5:** Coded project-related reflections.

No.	Author, year	Main/Focus study population	Challenges of/ within participatory activities	Ethical reflections	Methodological reflections	Influence of external events	Positionality and role of academic researchers
1	Afeworki et al. (46)	Migrants with a disability	Yes	Yes	Yes	Yes	Yes
2	Evangelidou et al. (52)	Vulnerable Migrants and refugees recently arrived (<5 Years in Europe)	Unclear	Yes	Yes	Yes	Yes
3	Geldermann et al. (45)	Migrants from a specific district	Yes	Yes	Yes	Unclear	Unclear
4	a) Herbert-Maul et al. (53) b) Fleuren et al. (54)	Women with low SES, migration background	Unclear	Unclear	Yes	Unclear	Unclear
5	a) Kieslinger et al. (55) b) Kordel et al. (56)	Refugees in rural areas	Yes	Unclear	Yes	Yes	Unclear
6	Koschollek et al. (57)	Members of local African communities	Unclear	Yes	Unclear	Unclear	Unclear
7	Perplies et al. (59)	Migrants from a specific district / with low SES	Yes	Unclear	Yes	Yes	Unclear
8	Pallasch et al. (59)	Migrants from Syria, Afghanistan	Yes	Unclear	Yes	No	No
9	a) Salman (60) b) Salman et al. (10)	Migrants from different countries (not specified)	Yes	No	Unclear	Yes	No
10	Samkangge- Zeep et al. (62)	Turkish migrants (born in Turkey/Turkish nationality)	No	No	Unclear	No	No
11	Sauer et al. (47)	Refugee minors living in long- term residence	Yes	No	Yes	Yes	Yes
12	Schön (63)	Migrants with a disability and their relatives / mostly mothers	Unclear	Unclear	Yes	Yes	Unclear
13	a) Von Unger (18) b) Von Unger et al. (64)	Migrants from African and European countries	Unclear	Unclear	Yes	Yes	Yes

**Table 6:** Overview of inductively coded subcategories and their examples^a^.

Categories of reflection	Coded subcategories	Examples of reflections
Challenges of/ within participatory activities	Collaboration-related challenges	Building and gaining trust (1, 3, 11, 13) Language diversity and linguistic barriers among academic and co-researchers (6, 8) Ensuring meaningful and continued engagement (8, 11)
	Structural and logistical challenges	Resource intensiveness of participatory approaches (3) Time constraints for repetitive field visits and long data collection sessions (6, 12)
Ethical reflections prior to doing participatory research ^b^	Intercultural and language sensitivity	Tailoring and translation of consent forms to different cultural and language groups (3, 4, 11, 12, 13)
	Participant support	Safe spaces for conducting emotionally challenging interviews (1) Appropriateness of the questions/research topic (1) Aftercare and post-interview participant well-being (1) Precautions for co-researchers with short-term legal status (12) Adequate compensation for co-researchers (13)
Methodological reflections	Sampling and recruitment	(Mis)trust in research and institutions by co-researchers (1, 5) Lack of connections and trusted networks to reach co-researchers (1) Additional resources required (time, personnel, representatives of the community) (9) Linguistic challenges between academic and co-researchers (4)
	Data collection	(Un)suitability of tools (5, 7) Linguistic challenges (challenges of working with interpreters) (1, 4, 5) Managing group dynamics (4, 5, 8, 11) Inclusion of caregivers or other trusted persons of the co-researchers (1)
	Data analysis	Triangulation of perspectives (13) Inconsistencies in data collected by co-researchers (1, 3)
Influence of external events on research project	Political and legal influences	Legal status and deportation obligations for peer researchers (8, 11) Changes in political guidelines (e.g., laws for sex workers) influenced participation (13)
	Research overlap	Over studied populations and similarities with other projects (6, 9)
Positionality and role of academic researchers	Differences in backgrounds and social identities	Knowledge hierarchies (6, 9, 10, 13) Language and cultural differences, affecting mutual understanding and trust (1, 2) Differences in social identities (11, 13)
	Authority as project leaders	Balancing the dual roles of project leadership and activity facilitation while adhering to participatory principles (2)
	Expectations	Managing expectations and desired impacts of academic and co-researchers (5)

a Examples in Table 5 include entries coded as “Yes” or “Unclear” in Table 4. “Unclear” indicates references where it did not become clear whether the reflections where theoretical or specifically project-related or the reflection was not explicitly stated.

b Only No. 5 mentioned the importance of ethical reflections throughout the project, yet did not specify them in relation to the project.

An observable pattern is the reference to external factors, impacting research processes, including changes in deportation laws (No. 8, 11) and sector-specific regulations (No. 13). This suggests that participatory research with migrant communities is shaped not only by internal project dynamics but also by broader political and legal environments. Reflections on collaboration and logistics, such as trust-building (No. 1, 3, 11, 13) and sustaining engagement (No. 8, 11) were also common. In contrast, considerations of positionality and power imbalances were less systematically discussed and limited to brief mentions.

Overall, the distribution of reflections indicates a focus on practical and contextual challenges, with less emphasis on ethical and positionality-related aspects, which mirrors the participation in specific research phases as described earlier (see c). While some projects were reflection-heavy (e.g., No. 1, 11), others contained little reflections (e.g., No. 4, 6, 10). Noteworthy is also the high number of codings as ‘unclear’. This coding was used when papers provided rich theoretical reflections in their introduction or theoretical framework but lacked clear connections to the actual reflexive activities within the project.

## Discussion

This scoping review mapped how participatory health research and promotion (PHR/P) with migrant communities in Germany is described in the scientific literature. In the following, key patterns and recurring gaps are discussed across the four analytical dimensions (a–d).

Our review revealed that PHR/P projects with migrant communities in Germany encompassed a diverse range of health-related and participatory-related objectives (a). Especially in projects identified as PHP, the extent to which participation was an explicit objective varied and appeared to function primarily as a methodological tool rather than as a guiding principle. This aligns with debates in PHR about the risk of instrumental participation and the importance of making participatory purposes explicit (6, 21, 32).

On who participated (b), projects consistently involved academic researchers and migrant co-researchers, with additional stakeholders variably included. These constellations appeared to enable access to diverse groups, but it also raises questions familiar in the literature about gatekeeping and knowledge hierarchies: stakeholder presence can open doors while also shaping who is seen and which forms of knowledge are legitimized (40, 41). Authorship patterns reinforced this concern as migrant co-researchers were less often visible as authors than other stakeholders, converging with prior reviews that showed limited crediting of co-researchers from marginalized groups (42, 43). This matters because authorship is both recognition and a public record of who is allowed to speak for the research.

For participation across phases (c), the drop-off after data collection was noticeable: publications most often reported involvement in operationalization and fieldwork, much less in conceptualization, analysis, or outputs. This finding aligns with broader critiques that participatory processes tend to be phase-bound in practice (44) and risk reproducing the hierarchies PHR/P sets out to unsettle. This is especially problematic when co-researchers are not consistently included in those phases, where meaning is negotiated and disseminated (6). The idea of optimum participation (31) is helpful here as it acknowledges limitations of participation. Our data, especially the omissions and unclear coded participation in early and late phases, suggests that such routes might have been taken, yet reasons for excluding participation during certain research phases should be more explicit.

Reflections on participatory processes varied greatly (d). Notably, reflections on methods and external influences were more frequently discussed than ethical concerns or researcher positionality, suggesting that while structural constraints are acknowledged, internal power dynamics within research teams are less reported. We also noted great variations in how reflexivity was integrated and reported across the studies reviewed. This disconnect led to some inconsistencies during our coding process as mentioned in the methods section, and required the refinement of coding categories to theoretical and project-related reflections. The noted gaps and differences in how reflexivity was reported may be partially influenced by the type of publication. Traditional research articles adhering to structured formats, such as introduction, background, methods, and results, often provided limited reflections on participatory processes, although this was not universally consistent [e.g., (45)]. In contrast, book chapters and anthologies tended to offer more detailed and nuanced accounts of these experiences [e.g., (46, 47)]. Moreover, we noticed that among those projects who had published more than one publication, different aspects or phases of the projects were focused upon in the different publications. This suggests that what is omitted in one publication may not necessarily be absent in practice, but rather a reflection of the constraints of scientific publishing.

### Implications

The findings of this review carry implications for the practice, publishing routines, and theoretical development of PHR/P with (and without) migrant communities. A key implication for practice concerns the operationalization of participation itself. While participation is a defining feature of PHR/P, our review demonstrates that migrants’ involvement across research phases often appeared limited. These patterns may reflect both structural constraints and varying interpretations of participatory principles (6, 44). We suggest more context-sensitive and flexible approaches to participation, grounded in ongoing negotiation and reflexivity on the constraints that shape migrants’ participation in participatory research (34, 48).

Regarding publishing routines, the review underscores the influence of publication formats on what is made visible. Journal articles, constrained by length and structure, tended to underreport reflexive and participatory dynamics. In contrast, alternative formats, such as anthologies or reports, offered more space for discussing team processes and tensions. This raises broader questions about how knowledge production is shaped by academic conventions (49). Promoting inclusive forms of authorship and offering explicit rationales when co-researchers are not named can support transparency, especially when anonymity or ethical concerns play a role. Additionally, expanding publishing standards to accommodate methodological reflections, e.g., via Supplementary materials or reflexivity statements, could help make participatory work more visible and learnable (50, 51).

Theoretically, the findings of this review do not imply a failure of participatory research itself but rather highlight the need for a more critical engagement with the conditions under which participation occurs and what is made visible in published accounts. They reinforce the existing demand for more differentiated understandings of participation, reflexivity, and power in PHR/P (6, 30, 33). A more explicit interrogation of how participation is defined, negotiated, and documented could help ensure that PHR/P does not merely include migrant communities but also critically reflects on the conditions that enable – or limit – their engagement. This is particularly relevant in contexts where questions of epistemic authority, selective inclusion, and representation intersect. Building on existing work (40, 43), further theorisation may help clarify how participatory principles can be meaningfully translated into practice.

### Strengths and limitations

This review contributes to the growing body of research critically examining participatory health research by offering a structured synthesis of how participation and reflexivity are enacted and reported in studies with migrant communities. It is, to our knowledge, the first review to focus specifically on the German context and synthesises a diverse and heterogeneous body of literature. Methodically, the combined approach of deductive and inductive coding categories allowed for a nuanced analysis of reflexivity- related aspects.

Several limitations must be acknowledged. First, by restricting the scope to studies from Germany, the transferability of findings to other contexts may be limited. Second, despite comprehensive search strategies, it is possible that relevant studies, particularly those published in formats not indexed in the selected databases or written in non-German or non-English languages, were excluded. In addition, we did not assess the methodological quality or health-related results of the included studies, as our focus was intentionally directed towards participatory processes rather than study outcomes or rigor, due to the heterogeneity of study designs and topics. Third, the review relied on what was explicitly reported in the publications. As many studies provided limited details on participatory processes, particularly in traditional journal articles constrained by word limits, the findings may not fully reflect the extent or nature of participation or reflexivity in practice. Lastly, while our coding process sought to ensure consistency, some subjectivity in the interpretation of reflexivity and participatory practices is unavoidable, particularly given the varied ways these concepts were reported across studies.

## Conclusion

This review mapped current descriptions of PHR/P with migrant communities in Germany, providing an overview of how participation and reflexivity are presented in published studies. While the included studies illustrate a broad thematic and methodological range, they also reflect recurring challenges in operationalizing participatory principles across research phases and documenting reflexive practice. The observed inconsistencies in reflexivity and its documentation emphasize the need for more structured yet adaptable approaches to reflect critically on power dynamics, roles, and methodologies. These findings contribute to a better understanding of the current state of the field and support further methodological development in how PHR/P with structurally marginalized groups is implemented and reported.

## Data Availability

The raw data supporting the conclusions of this article will be made available by the authors, without undue reservation.
