# Supplementary material for: Participatory health research and promotion with migrant communities in Germany: a scoping review
Source: Front Public Health. 2025 Sep 12;13:1585178. doi: 10.3389/fpubh.2025.1585178 (PMC12463829; doi:10.3389/fpubh.2025.1585178)
Supplement: Supplementary file 1 [file Data_Sheet_1.pdf]

**Coding Book Review Participation**

Note: The codebook also entails categories for coding that were not reported in the review.

| Category | Subcategories | Guiding questions | Characteristics |
|---|---|---|---|
| **General description** | Title of the study | What is the title of the study? | Please describe, including DOI |
| | Authorship | Who are named as authors? | 1 = Academic researchers<br>2 = Stakeholders from non-academic instituons<br>3 = Authors without instituional affiliation<br>4 = Co-researchers/Peer researchers<br>5 = Other |
| | | If 2 - 5, please specify | Please describe and mark in the text |
| | Type of publication | What type of publication is it? | 1 = Book chapter<br>2 = Article in collected work/compilation<br>3 = Article in peer reviewed journal<br>4 = Article in a non peer-reviewed journal |
| | Year of Publication | In which year was the study published? | Please describe and mark in the text |
| | Topic | What is the health-related topic of the project? | Please describe and mark in the text |
| | Type of project | What type of participatory approach does the publication report about? | 1 = Participatory health promotion<br>2 = Participatory health research |
| | Funding | Was the study or project sponsored by a third party? | 1 = Yes<br>2 = Not named<br>3 = No |

| | | | |
|---|---|---|---|
| | | If yes (1), which one? | Please name paragraph,<br>programme or institution |
| Study population | Included groups | Which study population is the focus of the project? | 1 = People with a migration background/migrant communities<br>2 = Children |
| | | If 1, which countries of origin do the persons come from? | Name countries of origin and exact migration background (if described) |
| | | If 2, what age group of children are involved?<br>Please specify (multiple answers possible): | 1 = Infants (0-5)<br>2 = Young children (6-9)<br>3 = Older children (10-12)<br>4 = Young teenagers (13-15)<br>5 = Older teenagers (16-18) |
| | | Are there other socio-demographic categories in the focus study population that are explicitly taken into account? | 1 = Yes<br>2 = Not named<br>3 = No |
| | | If yes (1), which one (s)? | 1 = Gender<br>2 = Age<br>3 = SES<br>4 = 1st generation of immigrants<br>5 = 2nd generation of immigrants<br>6 = legal status (e.g. refugees, asylum seekers)<br>6 = other (name) |
| | | If Gender (1), which one? | Please describe and mark in the text |
| | Excluded groups | Are certain groups explicitly excluded according to the description? | 1 = Yes<br>2 = Not named<br>3 = No |

| | | | |
|---|---|---|---|
| | | If yes (1), which one and for what reason? | Please describe and mark in the text |
| | Recruitment | How is the participating study population in focus recruited (snowball method, doctors, etc.)? | 1 = Stakeholder<br>2 = Institutions<br>3 = Snowball method<br>4 = Peers/ people from the community<br>5 = Other<br>6 = Not named |
| | | If peers/people from the community (4), please name/describe and mark in the text. | |
| **Introduction of participation as a concept and terminology** | Theoretical consideration of participation | Do the authors utilise theoretical models, concepts, frameworks for participation (e.g. stage model of participation, participatory health research model)? | 1 = Yes<br>2 = Not named<br>3 = No |
| | | If yes (1), which one? | Please describe and mark in the text |
| | | Are further theoretical models/theories introduced and explained (e.g. health competency, health literacy)? | 1 = Yes<br>2 = Not named<br>3 = No |
| | | If yes (1), which one? | Please describe and mark in the text |

| | | | |
|---|---|---|---|
| | Terminology | What terms do the authors use to describe the participatory study (multiple answers possible)? | 1 = Participatory health research (PHR)<br>2 = Community-based participatory research (CBPR)/Community-based participatory research<br>3 = Action Research<br>4 = Participatory health promotion<br>5 = Collaborative research<br>6 = Health services research<br>7 = Other<br>8 = None |
| | | If Other (7), which? | Please describe and mark in the text |
| | | How are the respective terms defined/introduced? | Please describe and mark in the text |
| | | How are the people involved who are not described as being academic researchers (multiple answers possible)? | 1 = Real world researchers<br>2 = Co-researchers<br>3 = Researchers from experience/Experts by experience<br>4 = Research team member<br>5 = Participants<br>6 = Peer researchers<br>7 = Other |
| | | If Other (7), which one? | Please describe |
| **Objectives and their effects** | Health-related objectives | Which health-related objectives does the project pursue (e.g. promoting physical activity, improving mental health, health literacy) (multiple answers possible)? | Please describe |
| | Participatory-related objectives | What are the overarching objectives of the study? (multiple answers possible) | 1 = Development of an intervention<br>2 = Exploration/generation of knowledge about challenges & opportunities of a health-related topic<br>3 = Development of a survey/research instrument (e.g. questionnaire)<br>4 = Behavioural change (e.g. increase in exercise in everyday life);<br>5 = Collect and strengthen resources (e.g. social networks, financial/material resources)<br>6 = Other<br>7 = Not named |

| | | | |
|---|---|---|---|
| | | If Other (6), please specify. | |
| | | Is there explicit description of the objectives related to participation (e.g. empowerment, representation, redistribution of power)? | 1 = Yes<br>2 = Not named<br>3 = No |
| | | If Yes (1), what are the objectives for the participants? | Please describe and mark in the text |
| | Effects | To what extent were these objectives defined with all groups/stakeholders? | 1= All goals were defined together<br>2 = Participatory researchers each have defined sub-goals<br>3 = Goals were discussed and could be processed/adjusted<br>4 = Goals were justified<br>5 = Targets have been set<br>6 = Not named<br>7 = Other |
| | | If No information (6) or Other (7), please specify. | |
| | | Is the achievement of the participatory objectives reported? | 1 = Yes<br>2 = Not named<br>3 = No |
| | | If Yes (1), how is the achievement of the participatory-related objectives reported? | Please describe and mark in the text |
| | | What impact do the authors describe in relation to the participatory objectives (e.g. degree of participation, networking, improvement of equal opportunities)? | Please describe and mark in the text |
| | | Are the interests/expected benefits of various stakeholders reported? | 1 = Yes<br>2 = Not named<br>3 = No |
| | | If Yes (1), please name and mark in the text. | |
| **Methodological approach** | Study design | Which study design was used? | 1 = Cross-sectional study<br>2 = Long-term/multiple |

| | | | |
|---|---|---|---|
| | | | surveys/pretest-posttest<br>3 = With control group<br>4 = Without control group<br>5 = Unclear<br>6 = other |
| | | If Unclear (5) or other (6), please describe and mark in the text. | |
| | Duration of study | In which period did the whole study take place? | Please name (in months) |
| | | In which period did data collection take place? | Please name (in weeks) |
| | Intervention | Was there an intervention related to the health goal (e.g. sports course to promote physical activity, nutrition course for weight reduction)? | 1 = Yes<br>2 = Not named<br>3 = No |
| | | If Yes (1), please describe and mark in the text. | |
| | | If several study populations participated in the study - who participated in the intervention? | Please describe and mark in the text |
| | | In which setting did the intervention take place? | 1 = Municipality/ community centre<br>2 = place of learning (school, university of applied sciences, community college)<br>3 = Workstation/workplace<br>4 = long-term residence (e.g. hospital, residential group, other forms of accommodation...)<br>5 = Other |
| | | If Other (5), please name and mark in the text. | |
| | Further measures | Have other activities been carried out to prepare or promote the project (e.g. training of participatory community staff/participants, information event on the study, relationship building through additional meetings)? | 1 = Yes<br>2 = Not named<br>3 = No |
| | | If Yes (1), please name and mark in the text. | |
| | Data collection | Which method(s) were used in the data collection (multiple answers possible)? | 1 = Written questionnaire/survey<br>2 = Interviews<br>3 = Focus groups |

| | | | |
|---|---|---|---|
| | | | 4 = Experiment<br>5 = Ethnographic methods<br>6 = creative methods (e.g. drawings, photos, theatre etc.)<br>7 = Mix of methods<br>8 = other |
| | | If Creative methods, mix of methods or other (6-8), which ones? | Please describe and mark in the text |
| | | If several study populations are participating in the study - who took part in which data collection? | Please describe and mark in the text |
| | | What types of knowledge were collected? | 1 = professional expertise<br>2 = academic/scientific knowledge<br>3 = Patient knowledge<br>4 = Network knowledge<br>5 = social and life-world knowledge<br>6 = non-biomedical, nonevidence-based health/specialist knowledge<br>7 = Other |
| | | If Other (7), please describe and mark in the text. | |
| | | How was the data of the focus study population collected (multiple answers possible)? | 1 = independently completed<br>2 = collected through interaction with another person (face-to-face, by telephone)<br>3 = Other |
| | | If Other (3), please describe and mark in the text. | |
| | | Who collected the data? (multiple answers possible) | 1 = academic/scientific researchers<br>2 = lifeworld/ peer/ co-researchers<br>3 = Other |
| | | If Other (3), please describe and mark in the text. | |
| | | In which setting did the data collection take place? | 1 = Municipality/ community centre<br>2 = place of learning (school, university of applied sciences, community college)<br>3 = Workstation/workplace |

| | | | |
|---|---|---|---|
| | | | 4 = long-term residence (e.g. hospital, residential group, other forms of accommodation...)<br>5 = Other |
| | | If Other (5), please name and mark in the text. | |
| | Linguistic materials | Were the communication materials (consent form, data collection, etc.) adapted to the focus study population? | 1 = Yes<br>2 = Not named<br>3 = No |
| | | To what extent were the communication materials (consent form, data collection, etc.) adapted to the focus study population? | 1 = Difficulty level/comprehensibility (e.g. easy language)<br>2 = written translation into the participants' native language<br>3 = Visualisations<br>4 = other persons present (e.g. translator, integration worker, etc.)<br>5 = Other<br>6 = Not or not named |
| | | If 1 - 5, please describe and mark in the text. | |
| | Data analysis | What methods were used to analyse the data? | 1 = Thematic/ content analysis<br>2 = Grounded Theory<br>3 = Statistical methods<br>4 = Other |
| | | If Other (4), please describe and mark in the text. | |
| **Research process** | Participation in phases of the research project | Do the authors differentiate between different types of involvement/participation in the study design? | 1 = Yes<br>2 = Not named<br>3 = No |
| | | If Yes (1), how are they described and defined? Please Describe and mark in the text. | |
| | | Are people in the key study population part of steering or decision-making bodies (e.g. advisory board, steering group, etc.)? | 1 = Yes<br>2 = Not named<br>3 = No |
| | | If Yes (1), how is this defined? Please describe and mark in the text. | |

| | | | |
|---|---|---|---|
| | | In which phases is the focus study population actively/participatively involved (e.g. other peers interviewed, involved in data analysis) (multiple answers possible)? | 1 = Recruitment of participants<br>2 = Creation of survey instruments<br>3 = Data collection<br>4 = Data analysis<br>5 = Publications, policy papers, other outputs<br>6 = Workshops, conferences to discuss/distribute the results<br>7 = Other (please specify) |
| | | For 1 - 7 please specify for each type of participation and mark in the text. | |
| | | In which phases does the degree of participation of the focus study population remain unclear (e.g. through passive constructions/descriptions)? | 1 = Recruitment of participants<br>2 = Creation of survey instruments<br>3 = Data collection<br>4 = Data analysis<br>5 = Publications, policy papers, other outputs<br>6 = Workshops, conferences to discuss/distribute the results<br>7 = Other (please specify)<br>8= None |
| | | From which phases of the research process was the focus study population explicitly excluded? | 1 = Recruitment of participants<br>2 = Creation of survey instruments<br>3 = Data collection<br>4 = Data analysis<br>5 = Publications, policy papers, other outputs<br>6 = Workshops, conferences to discuss/distribute the results<br>7 = Other (please specify)<br>8 = None |

| | | | |
|---|---|---|---|
| | | If there were explicit exclusions, on what grounds? | Please specify and mark in the text |
| | | In which phases are other study populations actively/participatively involved (multiple answers possible)? | 1 = Recruitment of participants<br>2 = Creation of survey instruments<br>3 = Data collection<br>4 = Data analysis<br>5 = Publications, policy papers, other outputs<br>6 = Workshops, conferences to discuss/distribute the results<br>7 = Other (please specify)<br>8 = None |
| | Output and processing of the results | Apart from this scientific publication being coded, are other outputs/processing of the results mentioned? | 1 = Yes<br>2 = Not named<br>3 = No |
| | | If Yes (1), please specify and mark in the text. | |
| | Effect | Are the effects of the participatory study design reflected? | 1 = Yes<br>2 = Not named<br>3 = No |
| | | If Yes (1), please describe and mark in the text. | |
| | | Were there any unintended effects (positive and negative)? | 1 = Yes<br>2 = Not named<br>3 = No |
| | | If Yes (1), please describe and mark in the text. | |

| | | | |
|---|---|---|---|
| **Reflection** | Ethics | Were concepts for the protection of participants (e.g. ethics application, data protection concept) developed in advance of the study? | 1 = Yes<br>2 = Not named<br>3 = No |
| | | If Yes (1), please describe and mark in the text. | |
| | | Were the concepts for protecting the participants critically reflected upon in the course of the study? | 1 = Yes<br>2 = Not named<br>3 = No |
| | | If Yes (1), please describe and mark in the text. | |
| | Methodology | Is the methodological approach reflected in the context of participation? | 1 = Yes<br>2 = Not named<br>3 = No |
| | | If Yes (1), please describe and mark in the text. | |
| | Context | Are (differences in) living conditions/life circumstances of all participants reflected? | 1 = Yes<br>2 = Not named<br>3 = No |
| | | If Yes (1), please name and mark in the text. | |
| | | Are the assumptions/attributions of the academic/scientific researchers reflected? | 1 = Yes<br>2 = Not named<br>3 = No |
| | | If Yes (1), please name and mark in the text. | |
| | | Is the influence of available resources (including experience) of the various participants reflected? | 1 = Yes<br>2 = Not named<br>3 = No |
| | | If Yes (1), please name and mark in the text. | |
| | | Is the influence of (external) events (e.g. pandemic, influence of external persons, departure of participants/key positions, loss of resources) reflected? | 1 = Yes<br>2 = Not named<br>3 = No |
| | | If Yes (1), please name and mark in the text. | |

| | | | |
|---|---|---|---|
| | Role of researchers in the research process | Do the academic researchers reflect on their role (role conflicts) with regard to interactions (resources, power, hierarchies, education, time) with the other participants (e.g. through a positioning/positionality statement)? | 1 = Yes<br>2 = Not named<br>3 = No |
| | | If Yes (1), please specify and mark in the text. | |
| | | Does the study address leadership, management and decision-making authority? | 1 = Yes<br>2 = Not named<br>3 = No |
| | | If Yes (1), please specify and mark in the text. | |
| | Work process | Is there reflection on the creation of a shared working atmosphere (building trust, conflict resolution, listening, joint learning...)? | 1 = Yes<br>2 = Not named<br>3 = No |
| | | If Yes (1), please specify and mark in the text. | |
| | | Is it reported if financial Compensation was made for co-researchers? | 1 = Yes<br>2 = Not named<br>3 = No |
| | | If Yes (1), please specify and mark in the text. | |
| | | Is there reflection on the development of the group/the group feeling for participants (e.g. empathy, sense of togetherness, appreciation)? | 1 = Yes<br>2 = Not named<br>3 = No |
| | | If Yes (1), please name and mark in the text. | |
| | Challenges and how to deal with them | Are challenges with participation reported? | 1 = Yes<br>2 = Not named<br>3 = No |
| | | If Yes (1), please name and mark in the text. | |

| | | | |
|---|---|---|---|
| | | Are unpredictability, chaos and the fact that these are part of the participatory research process reflected upon? | 1 = Yes<br>2 = Not named<br>3 = No |
| | | If Yes (1), please specify and mark in the text. | |
| | | Are lessons learnt/recommendations given for participatory projects? | 1 = Yes<br>2 = Not named<br>3 = No |
| | | If Yes (1), please name and mark in the text. | |
| | Literature references | Is further literature by the authors on the aforementioned project or other own writings related to participation listed? | 1 = Yes<br>2 = Not named<br>3 = No |
| | | If Yes (1), please name and mark in the text. | |
| **Comments and notes from the coders** | | | |
